# Identification of *Lactobacillus* strains from human mother milk and cottage cheese revealed potential probiotic properties with enzymatic activity

**DOI:** 10.1038/s41598-022-27003-2

**Published:** 2022-12-29

**Authors:** Abeer I. M. EL-Sayed, Aliaa M. El-Borai, Sara H. Akl, Samy A. EL-Aassar, Mohamed S. Abdel-Latif

**Affiliations:** 1 Botany and Microbiology Department, Faculty of Science, Damanhour University, El-Beheira, Egypt; 2 Botany and Microbiology Department, Faculty of Science, Alexandria University, Alexandria, Egypt; 3 Medical Laboratory Technology Department, Faculty of Applied Health Sciences Technology, Pharos University in Alexandria, Alexandria, Egypt

**Keywords:** Biochemistry, Biotechnology, Microbiology, Molecular biology

## Abstract

The main attempt of this study is to isolate, determine potential probiotic properties and enzyme production of some lactic acid bacteria (LAB). Among all isolates, two LAB strains isolated from human mother milk and cottage cheese revealed antimicrobial activity against some tested pathogenic strains. Both isolates inhibited all the tested pathogens except *Escherichia coli*. The two isolates were identified by morphological, biochemical properties and then by 16S rRNA gene sequencing technique as *Lactobacillus acidophilus* SAM1 and *Lactiplantibacillus plantarum* SAM2. Potential probiotic characters were investigated. Both strains survived in relatively low pH and high bile concentrations and were able to grow at 0.5% of pancreatin concentrations. Their growth decreased by increasing phenol from 0.2% till 0.5%. Both strains did not show hemolytic activity. Coaggregation potential was exhibited by the two strains against *Staphylococcus aureus* and *Candida albicans*. Hydrophobicity of *Lactobacillus acidophilus* SAM1 and *Lactiplantibacillus plantarum* SAM2, with ethyl acetate; were 88.1% and 82.8%, respectively. *Lactobacillus acidophilus* SAM1 was susceptible to Ampicillin, Penicillin, Erythromycin, Ciprofloxacin and Tetracycline; on the contrary, it resists Vancomycin and Cefoxitin; while *Lactiplantibacillus plantarum* SAM2 resists all tested antibiotics. Maximum growth was achieved using glucose as a carbon source and yeast extract as nitrogen source for both strains; however, glucose is the most preferred carbon source for microorganisms and it prevents the uptake of carbon from other sources like yeast by catabolite repression mechanism. *Lactobacillus acidophilus* SAM1 produces lipase enzyme, while *Lactiplantibacillus plantarum* SAM2 produces amylase and protease.

## Introduction

Nowadays, popular functional foods as dairy products are enriched with appropriate probiotics and prebiotics or their symbiotic combinations; also these are known to be an excellent chance to improve consumer's health^1,2^.

From the most frequently used probiotics are bacteria belonging to genus *Lactobacillus* which is successfully incorporated in functional dairy products. Also, It was proven that a daily dose of 10^9^ viable CFU/mL or g of a dairy product are required for consumption to ensure their health benefits on the consumer^3^. Health benefits of probiotics include prevention and treatment of diarrhea, lactose intolerance, modulation of gut microbiota, immunomodulation, alleviation of allergies and atopic diseases^4^.

However, probiotics vary widely according to their beneficial effects on the host. These variations exist at genus and species levels. Therefore, the beneficial effects that related to strain of a specific genus or species might not basically be a representative figure to alternative members of same genus/species. Accordingly, every individual probiotic candidate has to be experimentally investigated separately^5^.

Lactic acid bacteria (LAB) as *Lactobacillus*, *Bifidobacterium*, *Streptococcus* and *Enterococcus* species are widely used as probiotics, as they were noted to reduce the symptoms of diarrhea, inflammatory bowel disease and irritable bowel syndrome; these LAB must tolerate acids, bile, pancreatic digestive enzymes, and finally adhere to intestinal epithelium in colon^6,7^. Growth of LAB is strongly affected by fermentation conditions, including, pH, medium components. Thus, type of growth medium (MRS, M17 and skim milk) is critical for bacterial growth^7^.

*Lactiplantibacillus plantarum* species shows various biological effects, as antitumor, anticoagulant, antiviral, immunomodulatory, anti-inflammatory, anti-diabetic and anti-oxidative, consequently; it is taken by children and immunocompromised individuals^7^. *Lactobacillus acidophilus* is a commercial probiotic affects human ecosystem due to bacteriocins production and widely used in dairy industry to obtain high-quality fermentation products^8^.

The main objective of this study is to identify and examine the probiotic properties of LAB, such as acid tolerance, hydrophobicity, auto-aggregation, coagulation, bile salts and antibiotic sensitivity; also, evaluating their enzyme production and studying different carbon and nitrogen sources for maximum LAB growth.

## Materials and methods

### Bacterial isolation and culture media

Lactic acid bacteria were isolated from human mother milk, and cottage cheese, yogurt, boiled and unboiled milk, and pickles; all collected from different local dairy product markets.

Informed consents were obtained from all recruited individuals. Also, ethical approval for the study was obtained from the local ethical committee of the Faculty of Science, University of Alexandria, Egypt. All methods were carried out in accordance with relevant guidelines and regulations.

Sterile bottles and bags were used to collect samples, to be stored in an ice box until transferred to laboratory for further examinations. One milliliter of each sample was serially diluted using sterile saline solution and spread over selective medium: De Mann, Rogosa Sharpe (MRS) with the following composition (g/l): peptone, 10; beef extract, 10; yeast extract, 5; glucose, 20; tween 80, 1; ammonium citrate, 2; sodium acetate, 5; magnesium sulphate, 0.10; di-potassium phosphate, 2 and agar, 10. The medium pH was adjusted at 6.2 ± 0.2, and then incubated at 37 °C for 24 h^9^. For obtaining pure culture, separate and morphologically different colonies were isolated and, immediately, sub-cultured on freshly prepared MRS agar plates. All experiments and cultures were done in completely sterile conditions to avoid any fungal contaminations.

### Morphological, physiological and biochemical characterization of LAB isolates

Preliminary identification of the bacterial isolates was carried out by colony morphology, cultural characteristics, and microscopic observations. The isolates that were identified as Gram-positive, non-spore forming, and catalase-negative were selected and screened by physiological and biochemical tests, which include; catalase test, oxidase test, indole test, methyl red test, Voges Proskauer (VP) test, citrate utilization, salt tolerance, triple sugar iron test (TSI), arginine dehydrolase, and sugar fermentation test using three different sugars (glucose, lactose and sucrose)^10^. Isolates were also examined for growth at different temperatures (10, 30 and 45 °C), for motility and endospore formation. Among all collected samples, colonies which were identified as LAB were isolated only from human mother milk and cottage cheese. Then, LAB isolates were stored in 20% (v/v) glycerol at − 20 °C till further analysis.

### Molecular identification

The most promising isolates which achieved probiotics properties were identified by 16S rRNA gene sequencing analysis by GACT Company-Germany using universal primers; Forward: 5-AGA GTT TGA TCC TGG CTC AG-3 and Reverse: 5-GGT TAC CTT GTT ACG ACT T-3. PCR program was set according to the following cycling parameters: 3 min of initial denaturation at 95 °C, with 35 cycles of 30 s at 95 °C (denaturation), 30 s at 52 °C (annealing) and 2 min at 72 °C (extension), with a final extension at 72 °C for 10 min. NCBI tools as blast analysis and phylogenetic tree method were applied for the providing sequence and deposited to GenBank.

### Scanning electron microscope (SEM)

Bacterial cells were harvested, rinsed with phosphate buffer and fixed with 2% glutaraldehyde before being treated by 1% osmium tetraoxide treatment. After completion of fixation, samples were rinsed in buffer solution and dehydrated in ascending order of ethanol concentrations. The samples were thoroughly dried in a critical point drier before being gold-coated in a JEOL-JFG1100 E ion-sputter-coater. Specimens were examined in the Electron Microscope Unit of the Faculty of Science, Alexandria University, using a JEOL-JSM 5300 scanning microscope operating at 20 kV with a beam specimen angle of 45 °C.

### Antimicrobial activity

Antimicrobial activity of isolates against pathogenic strains was assessed using agar well diffusion method^11^. 100 μl of pathogenic bacteria as *Escherichia coli*, *Staphylococcus aureus*, *Pseudomonas aeruginosa* and *Candida albicans* (Department of Medical laboratory technology, Faculty of Allied Medical Science, Pharos University in Alexandria), was mixed with soft agar and overlaid on Muller Hinton Agar (MHA). Wells were made on MHA plates using sterile cork borer and 100 μl of overnight LAB isolates culture was poured in them. Plates were allowed to dry and incubated at 37 °C for 24–48 h, and then the presence of the inhibition zone was detected and measured.

### Screening of LAB for potential probiotic characters

#### Acid and bile salt tolerance

Bacterial isolates from 24 h old culture were used to inoculate (1%, v/v) MRS broth adjusted at pH 2, 3, 4, 5, and 6; and to 0.2, 0.4, 0.6, 0.8, 1, 2 and 3% of bile salts then incubated at 37 °C for 48 h. Optical density (OD_600nm_) was measured. Control cultures were grown on MRS broth without bile salts and pH^12,13^.

#### Effect of pancreatin and phenol

Bacterial isolates from 24 h old culture were inoculated in MRS broth containing 0.5% of pancreatin (Sigma-Aldrich) and cultured at 37 °C for 24 h and OD_600nm_ was measured. The tolerance to phenol was tested by inoculating 100 ml of 24 h old culture into MRS broth containing 0.2% and 0.5% phenol and measuring the OD_600nm_ after 24 h^13–15^.

#### NaCl tolerance

A 24-h-old bacterial culture was used to inoculate MRS broth containing NaCl (from 1 to 7%, w/v) and incubated at 37 °C for 48 h before measuring OD_600nm_^16^.

#### Hemolytic activity

Hemolytic activity was assessed by inoculating the 24 h old culture on blood agar plates [containing 5% (w/v) sheep blood] and incubated at 37 °C for 48 h and hemolysis zones were observed^17^.

#### Auto-aggregation and co-aggregation property of isolates with pathogens

To investigate the auto-aggregation property of bacteria, cells were harvested (4025 g, 10 min), the pellets were washed and suspended in phosphate-buffered saline (PBS). A vortex was used to mix a 4 ml suspension for 15–30 s. After varying time intervals, 0.1 ml from the top layer of cell suspension was taken to 3.9 ml of PBS in a new tube, mixed gently, and OD_600nm_ was measured^18^. Auto-aggregation percentage (AAg %) was expressed by:

$${\text{AAg}}\% = \left[ {1 - \left( {\frac{{A_{t} }}{{A_{0} }}} \right) \times 100} \right]$$

where A_0_ and A_t_ represent the absorbance at 0 h and after time interval of 1, 2, 3, 4 and 24 h, respectively^18^.

The property of co-aggregation was investigated. LAB and tested pathogens were cultured in MRS broth and nutrient broth, respectively, for 18 h at 37 °C to attain viable counts of approximately 10^8^ CFU/ml (A_600_ 0.85–0.9). 2 ml of individual LAB and pathogen were vortexed for 10 s at 37 °C. 0.1 ml of the top layer of the suspension was removed to a new tube filled with 3.9 ml of PBS and the absorbance (A_600_) was taken. Controls were set up simultaneously as 4 ml of the individual bacterial cell suspension^18^. The co-aggregation was calculated by;

$${\text{Coaggregation }}\;{\text{(\% ) = }}\frac{{\left[ {\frac{{\text{Apat + Aprobio}}}{2} - {\text{A(pat}} + {\text{probio}})} \right]}}{{\text{(Apat + Aprobio)/2}}} \times 100$$

where A_pat_ and A_probio_ represent absorbance (A_600_) of the individual bacterial suspensions in control tubes, and A _(pat + probio)_ mix represents the absorbance of the mixed bacterial suspension at different time intervals.

#### Hydrophobicity property

Hydrophobicity was measured using xylene, chloroform, and ethyl acetate. LAB isolates were cultivated in MRS broth for 18 h, and cells were harvested by centrifugation. Cell pellets were rinsed twice with Ringer solution (6 % NaCl, 0.0075 % KCl, 0.01 % CaCl_2_ and 0.01 % NaHCO_3_) and suspended in the same solution then A_580 nm_ was measured; using Ringer solution as blank. From the suspension; 1.5 ml was vortexed for 2 min with solvent (xylene, chloroform, or ethyl acetate) by a ratio of 1:1. After 30 minutes of incubation, the aqueous phase was separated carefully, from each solvent, and its OD_580_ nm was measured^18^. Hydrophobicity% was calculated by:

$$\mathrm{Hydrophobicity} \%=\frac{\mathrm{Abs}\left(\mathrm{initial}\right)-\mathrm{Abs}(\mathrm{solvent})}{\mathrm{Abs}(\mathrm{initial})} \times 100$$

where Abs (initial) represent initial absorbance (A_580nm_), and Abs (solvent) represent the absorbance in the presence of solvent.

#### Antibiotic susceptibility

Antibiotic susceptibility was assessed by disc diffusion sensitivity method; inoculating 1% of bacterial suspension of OD (0.881) on MHA plates by swabbing technique. Antibiotics discs (Ampicillin; 10 μg/ml, Penicillin; 10 μg/ml, Erythromycin; 15 μg/ml, Chloramphenicol; 10 μg/ml, Tetracycline; 30 μg/ml, Vancomycin; 30 μg/ml, Cefoxitin; 30 μg/ml,) were deposited and incubated at 30 °C for 24 h. By referring to the inhibitory zone around antibiotic discs, susceptibility breaking-point to various antibiotics was examined^19^.

#### Enzymatic activity

To detect amylase, protease and lipase activity the culture was inoculated into appropriate media and the zone of clearance was determined. For amylase activity, the bacterial strains were spread into modified MRS media (0.5% peptone, 0.7% yeast extract, 0.2% NaCl, 2% starch, and 1.5% agar) supplemented with 0.25% of starch. After incubation; the zone of clearance was identified by applying Gram’s iodine as detecting agent^20^. For protease activity, 50 ml of cell free extract was inoculated onto agar medium containing skimmed milk (1%) and incubated for 48 h. After incubation; the zone of clearance was observed and measured^21^. The activity of lipase was also measured in a medium containing olive oil (1%). MRS broth containing olive oil (1%) and Arabic gum was inoculated with 50 ml of cell free supernatant (1%). The zone was observed by adding phenol red after 48 h of incubation^22^. Qualitative extracellular enzyme activity was observed by the appearance of halo diameter in mm around the colony, which was presented as scores as follows: nil (no halo); +, low (1–4 mm halo); ++, intermediate (5–8 mm halo); +++, high (9–12 mm halo); ++++, very high (≥ 13 mm halo)^23^.

#### Influence of carbon and nitrogen sources on growth

The main sugar in original MRS medium [glucose (dextrose)] was substituted with equivalent weight of different carbon sources as sucrose, maltose, starch, fructose, mannitol and without main carbon source. 50 ml of medium in each flask was inoculated with an overnight culture of the probiotic strain 2% (v/v) and incubated at 37 °C for 24 h., and then OD_600 nm_ was measured.

The main nitrogen sources in original MRS medium; were substituted in an equivalent weight with different nitrogen sources as; beef extract, peptone, yeast extract, ammonium chloride, ammonium sulphate, casein and sodium nitrate. 50 ml medium in each flask was inoculated with an overnight culture of the probiotic strain 2% (v/v) and incubated at 37 °C for 24 h, and then OD_600nm_ was measured.

### Statistical analysis

All the experiments were performed in triplicates, and the data were expressed as the means ± standard deviation of three independent replicates. Comparison between the means of the LAB isolates was analyzed using one-way ANOVA with Fisher’s least significant difference test. Differences were considered significant at P < 0.05. The gathered data were analyzed using IBM SPSS version 24.0.

## Experimental results

### Morphological, physiological and biochemical characterization of LAB isolates

Among all collected samples, only two LAB isolates were identified; one from human mother milk (*Lactobacillus acidophilus* SAM1) and one from cottage cheese (*Lactiplantibacillus plantarum* SAM2). They revealed antimicrobial activity and have probiotic properties. Morphologically, colonies were circle, raised, smooth with entire margin, creamy, and slightly translucent. Microscopically, the strains were identified as Gram positive, rod-shaped, arranged in pairs or forming chains, non-motile, and non-spore former. Concerning the biochemical and physiological features, the two strains were catalase, oxidase, indole, VP, and arginine negative, and positive for methyl red only by *Lactobacillus acidophilus* SAM1, while in the citrate utilization test only *Lactiplantibacillus plantarum* SAM2 was positive. Both, can grow in a medium containing 2% and 4% NaCl (as explained later, in NaCl tolerance) and at 30–45 °C. Each strain can ferment different sugars (glucose, lactose and sucrose) with acid production, however, gas production was only indicated with *Lactobacillus acidophilus* SAM1. In the Triple Sugar-Iron (TSI) test; the two strains can change the media colour from orange to yellow; meaning that sugar fermentation took place (Supplementary material, Table 1).

### Molecular identification

**Figure 1:**
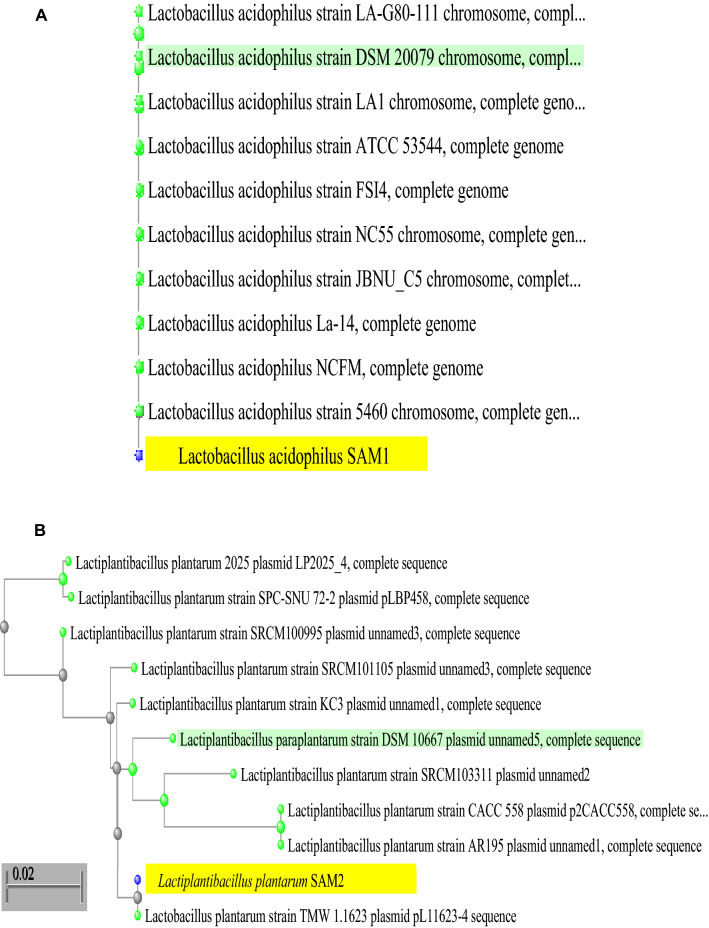
The phylogenetic tree of identified strains according to the partial sequence of 16S rRNA; (**A**) *Lactobacillus acidophilus* SAM1, (**B**) *Lactiplantibacillus plantarum* SAM2.

The two isolates were identified by 16s rRNA sequencing method. The similarity was 100% for the two selected predominant strains*.* Nucleotide sequence was submitted to GenBank sequence database, and given accession numbers which are ON495959 and ON527739 for *Lactobacillus acidophilus* SAM1 and *Lactiplantibacillus plantarum* SAM2, respectively (Fig. 1A,B).

### Scanning electron microscope (SEM)

**Figure 2:**
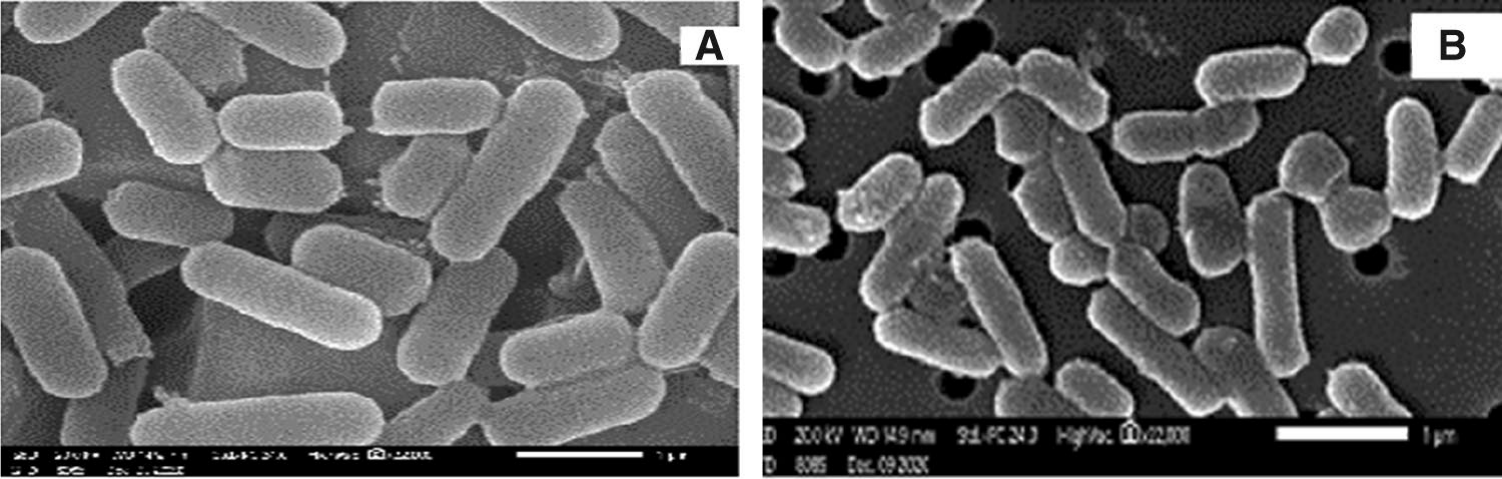
Scanning electron micrographs of cells; (**A**) overview of *Lactobacillus acidophilus* SAM1, (**B**) overview of *Lactiplantibacillus plantarum* SAM2.

Shape and arrangements of the two target strains, *Lactobacillus acidophilus* SAM1 and *Lactiplantibacillus plantarum* SAM2, were observed by SEM. The cells appear as long rod shaped attached in pairs and sometimes form chains (Fig. 2).

### Antimicrobial activity

*Lactobacillus acidophilus* SAM1 and *Lactiplantibacillus plantarum* SAM2 were examined for their antimicrobial activity. It was noticed that both strains inhibited the growth of most of the tested pathogens with inhibition zone of 2 ± 0.21, 1.8 ± 0.183, and 1.5 ± 0.136 cm against *Candida albicans, Staphylococcus aureus, and Pseudomonas aeruginosa,* respectively*;* by *Lactobacillus acidophilus* SAM1, and 1.8 ± 0.164, 1.7 ± 0.113, and 1.3 ± 0.130 cm against the same tested pathogens for *Lactiplantibacillus plantarum* SAM2. While *E. coli,* was resistant to the both strains as shown in Table 1.

**Table 1:** Antimicrobial activity of the LAB strains.

Strains	Indicator microorganisms and zone of inhibition (cm)			
	*C. albicans*	*S. aureus*	*P. aeruginosa*	*E. coli*
*Lactobacillus acidophilus* SAM1	2 ± 0.21	1.8 ± 0.183	1.5 ± 0.136	–
*Lactiplantibacillus plantarum* SAM2	1.8 ± 0.164	1.7 ± 0.113	1.3 ± 0.130	–

### Screening of LAB for potential probiotic characters

#### Acid and bile salt tolerance

**Figure 3:**
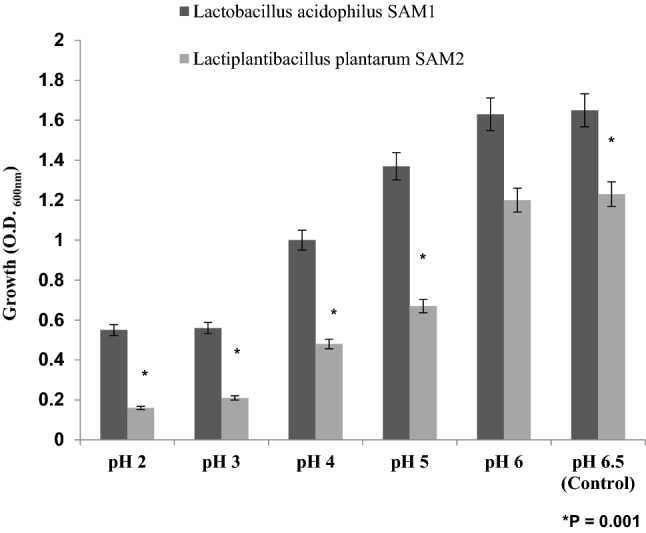
Acid tolerance of the LAB strains.

Growth of *Lactobacillus acidophilus* SAM1, significantly, increased gradually from pH 2 till reaching pH 6.5 with O.D. about 1.65 ± 0.110 (P = 0.001). The same growth increasing trend was noticed for *Lactiplantibacillus plantarum* SAM2 which rose gradually till pH 6.5 giving O.D. about 1.23 ± 0.082 (P = 0.001). That is meaning; both LAB strains showed a remarkable acidic tolerance pattern, as indicated in Fig. 3.

**Figure 4:**
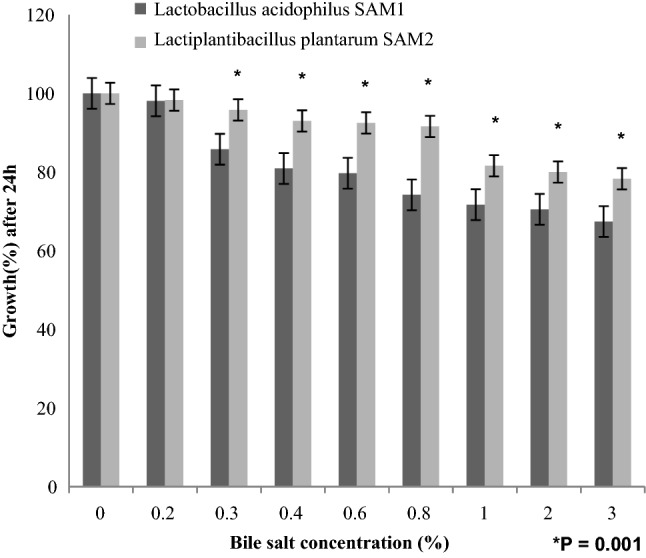
Bile salt tolerance of the LAB strains.

The present study also revealed bile salt tolerance at different bile salt concentrations for both LAB strains. The experiment reported a significant higher survival rate for *L. plantarum* SAM2, in 0.3% bile, with a marked growth rate of 78.3%; than that of *L. acidophilus* SAM1, which gave a viability rate of 67.4%; after 24 h incubation at 37 °C (P = 0.001). By increasing concentration of bile salts, the growth rate was, significantly, reduced (P = 0.001) (Fig. 4).

#### Effect of pancreatin and phenol

The present study reported a significant growth for both strains in 0.5% pancreatin, and the measured O.D. were 1.78 ± 0.148 and 1.77 ± 0.126, for *L. acidophilus* SAM1 *and L. plantarum* SAM2, respectively.

**Figure 5:**
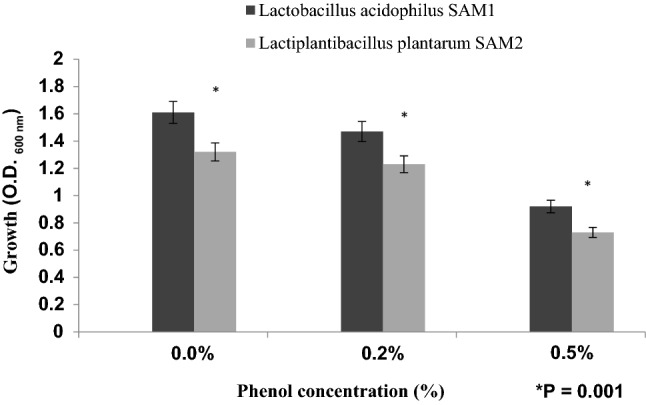
Effect of phenol concentrations on the growth of LAB strains.

There was a good tolerance of tested strains toward phenol; even if the growth in presence of phenol was lower than in MRS broth (zero phenol) for 24 h incubation. Viability rate of the two LAB strains was, significantly, decreased gradually by increasing phenol concentration (P = 0.001). However, *Lactobacillus acidophilus* SAM1 showed a higher phenol resistance in comparison with *Lactiplantibacillus plantarum* SAM2 (Fig. 5).

#### NaCl tolerance

**Figure 6:**
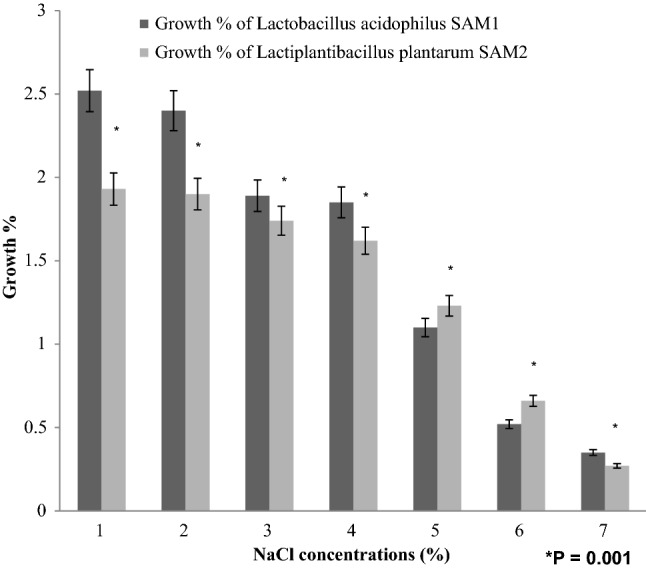
Effect of NaCl concentration on the growth of LAB strains.

Both LAB strains showed tolerance to different NaCl concentrations from 1 to 7% with a gradual decline in the viability. No viability was detected for both LAB strains by increasing NaCl concentration above 7%. Maximum growth was measured for *Lactobacillus acidophilus* SAM1 by O.D. equal to 2.52 ± 0.194 at 1% NaCl concentration; also, the growth for *Lactiplantibacillus plantarum* SAM2 was measured to be with O.D. equal to 1.93 ± 0.148 (Fig. 6).

#### Hemolytic activity

*Lactobacillus acidophilus* SAM1 and *Lactiplantibacillus plantarum* SAM2, exhibited γ-hemolytic activity (no hemolysis) when cultivated on blood agar (Supplementary material, Fig. 1).

#### auto-aggregation and co-aggregation property

**Figure 7:**
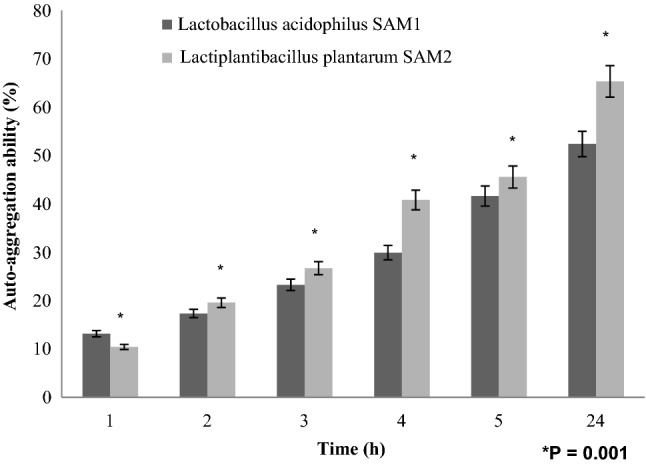
Auto-aggregation ability (%) of LAB strains.

The two LAB strains showed good auto-aggregation ability as represented in Fig. 7. Auto-aggregation property increased gradually with different incubation time till reaching remarkable auto-aggregation ability after 24 h at 37 °C, which were 52.39 and 65.32 for *L. acidophilus* SAM1 and *L. plantarum* SAM2, respectively.

**Figure 8:**
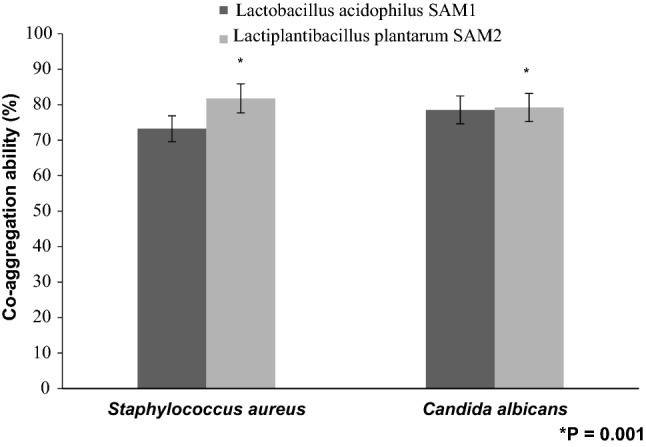
Co-aggregation ability (%) of LAB strains against pathogens.

Marked co-aggregation potential was also exhibited by the two LAB strains against the tested pathogens *Staphylococcus aureus* and *Candida albicans*. The co-aggregation abilities that detected for *L. acidophilus* SAM1 and *L. plantarum* SAM2 were 73.23% and 81.78%, respectively against *S. aureus*, and showed 78.45% and 79.22%, respectively toward *C. albicans* after 24 h of incubation at 37 °C (Fig. 8).

#### Hydrophobicity property

**Figure 9:**
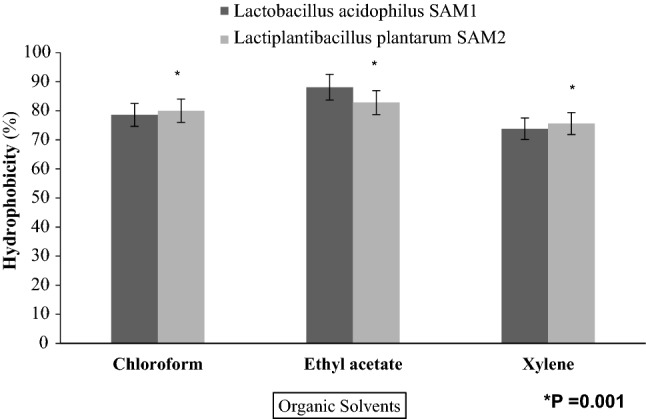
Hydrophobicity (%) of LAB strains with different organic solvents.

The maximum hydrophobicities of *L. acidophilus* SAM1 *and L. plantarum* SAM2 were found to be 88.1% and 82.8% with ethyl acetate, respectively. Additionally, marked hydrophobicities were also detected for both *L. acidophilus* SAM1 *and L. plantarum* SAM2 with chloroform and xylene, but lower than those for ethyl acetate, as shown in Fig. 9.

#### Antibiotic susceptibility

Results showed that *L. acidophilus* SAM1 was susceptible to all tested antibiotics; Ampicillin and Penicillin (from the β-lactam group of antibiotics), Erythromycin (from the macrolide group), Ciprofloxacin (from quinolones), and Tetracycline. A remarkable resistance of this strain was observed toward Vancomycin and Cefoxitin. However, *L. plantarum* SAM2 showed good resistance to all tested antibiotics; that was noticed by the absence of inhibitory zone around antibiotic discs (Supplementary material, Table 2).

#### Enzymatic activity

The extracellular lipase production by *L. acidophilus* SAM1 was observed by the zone of clearance measuring 11 mm around the colony, when treated with phenol red. *L. plantarum* SAM2 exhibited production ability for both protease and amylase enzymes with a halozones of 4 and 3 mm, respectively, when using skimmed milk in the production medium for protease detection and iodine for amylase detection (Table 2).

**Table 2:** Extracellular enzyme production of the LAB strains.

Strains	*L. acidophilus* SAM1	*L. plantarum* SAM2
**Enzymes**		
Protease enzyme	–	+
Amylase enzyme	–	+
Lipase enzyme	+++	–

Qualitative extracellular enzyme activity was observed by the appearance of halo diameter in mm around the colony, which was presented as scores as follows: nil (no halo); +, low (1–4 mm halo); ++, intermediate (5–8 mm halo); +++, high (9–12 mm halo); ++++, very high (≥ 13 mm halo).

#### Effect of carbon and nitrogen sources on LAB growth

**Figure 10:**
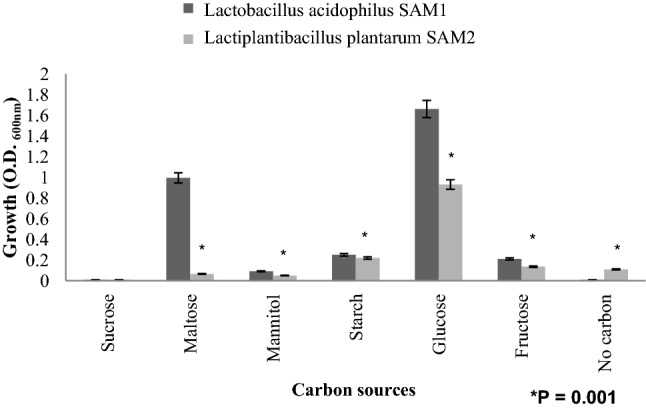
Influence of different carbon sources on growth of LAB strains.

The effect of different carbon sources on the growth rate of *L. acidophilus* SAM1 and *L. plantarum* SAM2 was examined*.* The results in Fig. 10 indicated that the original carbon source (glucose) has a marked inducing effect on the growth of *L. acidophilus* SAM1 and *L. plantarum* SAM2, with O.D. equal to 1.66 ± 0.111 and 0.93 ± 0.066; respectively. Also, it has been shown that glucose inducing effect on the growth of the two *LAB* strains was significantly higher, as compared to other carbon sources (P = 0.001). On the other hand, maltose triggered a very significant good growth of *L. acidophilus* SAM1 when compared to *L. plantarum* SAM2, with O.D. equal to 0.994 ± 0.090 and 0.066 ± 0.007; respectively.

**Figure 11:**
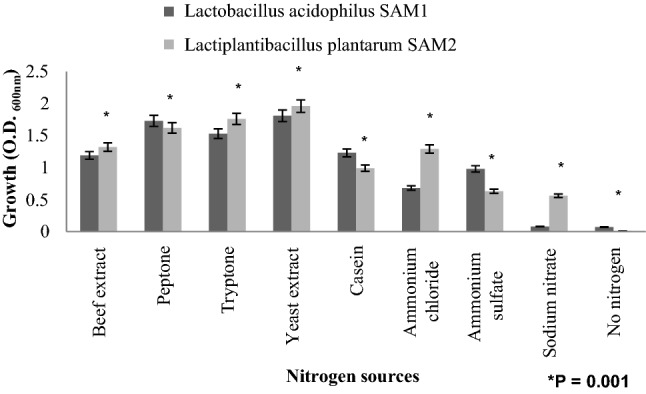
Influence of different nitrogen sources on growth of LAB strains.

The effect of different nitrogen sources on growth rates of *L. acidophilus* SAM1 and *L. plantarum* SAM2 was also investigated. The best used nitrogen source was yeast extract as illustrated in Fig. 11. Yeast extract showed a significant highest growth rates among other nitrogen sources, with O.D. equal to 1.81 ± 0.139 and 1.96 ± 0.131 for *L. acidophilus* SAM1 and *L. plantarum* SAM2; respectively (P = 0.001).

## Discussion

Lactic acid bacteria were isolated from different sources, and their probiotic potential was examined. Only two strains were identified as *Lactobacillus acidophilus* SAM1 (from human mother milk) and *Lactiplantibacillus plantarum* SAM2 (from cottage cheese). The two strains were Gram-positive bacilli, non-spore forming, and tested negative for catalase, oxidase, indole, MR-VP negative and positive for glucose and sucrose which is similar to that reported by Tallapragada et al.^24^.

A promising probiotic bacterial isolate should tolerate low pH in the GIT environment^25^. *Lactobacillus acidophilus* SAM1 and *Lactiplantibacillus plantarum* SAM2 showed maximum tolerance; due to the great ability of *lactobacilli* to adapt for acid during their growth in MRS broth^26^.

Findings of Dave and Shah (1998) and Liong and Shah (2005) stated that probiotic standard cultures could resist acidic pH value (pH 3)^27,28^. While Todorov et al.^29^ reported that maximum growth of *L. plantarum* ST16Pa was noticed at pH 4 and 5. *L. plantarum, L. rhamnosus, L. pentosus, and L. paracasei* achieved low growth at pH 2 and 3^29^. Previous researchers mentioned that different concentrations of hydrogen ion have had a strong effect on the bacterial survival^30^. Another study has reported that acids such as hydrochloric acid found in human stomach could disrupt the cell biomolecules, such as fatty acids, proteins and DNA^31^. Acidic pH environments may inhibit metabolism and reduce the viability of Lactobacilli. Other studies have shown that upon exposure to gastric acid with a pH of 2; a significant reduction in the number of bacteria could be recognized^31,32^. Alameri et al.^33^ mentioned that; probiotics should possess good resistance toward bile salts in order to survive in the human GIT. Therefore, high survival percentages indicate good bile salt tolerance. Bile plays an important role in the specific and nonspecific intestinal defense mechanism of the gut, and the severity of its inhibitory action is primarily determined by bile salt concentrations^34^. In this study, *Lactobacillus acidophilus* SAM1 exhibited a moderate tolerance to bile salt while *Lactiplantibacillus plantarum* SAM2 showed a significant tolerance; these finding were in accordance by those of Jamaly et al.^35^ who reported that *L. plantarum*, *L.paracasei* and *L. brevis* could resist 0.3% Ox-bile, additionally Kim et al.^36^ listed that *L. gasseri*, *L. acidophilus* and *L. brevis* could grew when exposed to 1% bile and more. Similar results have been obtained by Yasmin et al.^30^. The potential of the studied strains differs from that of other strains in acid and bile tolerance, that could be due to differences in cell wall structure as suggested by Conway et al.^37^. However, Boke et al.^38^ mentioned that, lactic acid bacterial exopolysaccharides should be used to protect microbial cells from acidic pH and bile salts. Pancreatic enzymes are secreted into the small intestine via the pancreatic duct and are involved in the digestion of proteins, carbohydrates, and fats in foods. A criterion for probiotic bacteria selection is the ability to tolerate the presence of pancreatic enzymes, which are present in 0.5% of the human gastrointestinal tract. Those findings were in accordance with our results that showed the ability of *Lactobacillus acidophilus* SAM1 and *Lactiplantibacillus plantarum* SAM2 to survive at 0.5% pancreatin, and also supported by other studies which proved that many *Lactobacillus* strains showed strong growth at 0.5% of pancreatin^14,39^. Remarkable decline was noticed in the growth rate of *Lactobacillus acidophilus* SAM1 and *Lactiplantibacillus plantarum* SAM2 by increasing the phenol concentration. Phenols can be produced in the intestine due to bacterial deamination of aromatic amino acids derived from dietary and endogenous proteins, these compounds have been shown to have bacteriostatic properties in vitro as mentioned by Suskovic et al.^40^. Additionally, Xanthopoulos et al.^41^, noticed that a bacteriostatic activity in many microorganisms was mentioned when 0.4% phenol existed. Also Raja et al. (2009) and Hoque et al. (2010) listed that isolated *Lactobacillus *spp. could resist the inhibitory effect of 0.4% phenol^42,43^. It has been known that LAB strains are capable of tolerating high salt concentrations. Because, salt has a metabolic role in LAB which leads to lactic acid production that, further, inhibits the growth of unwanted microorganisms. NaCl in medium forces the bacterial cells to lose their turgor pressure which, in turn, affects their enzymes and water activities. Consequently, LAB neutralizes that effect by inducing osmolytes to regulate the inside and outside pressure^13^. Similarly, our findings showed that the growth of the two isolated strains decreased gradually by increasing the concentration of NaCl. Jeronymo-Ceneviva et al.^44^ stated that *Lactobacillus* strains isolated from dairy product showed survival in presence of NaCl (1–7%), proving their high sodium chloride tolerance. Hemolysis is a critical virulence factor for pathogenic microorganisms. Hemolytic activity of the two strains was investigated in the current study. Results showed ɤ-hemolysis (non-hemolytic) which considered a safe prerequisite for the selected probiotic strain as stated by Araya^45^. Similar observations have been reported by Maragkoudakis et al.^39^ in which; *Lactobacillus* species isolated from dairy products have been shown to be non-haemolytic. On the contrary, another study reported only few LAB isolates have shown hemolytic activity^46^. Aggregation between microorganisms of the same strain [auto-aggregation (AAg)] or between genetically different strains (co-aggregation) is of a great significance in numerous ecological niches, especially in human intestine^4,5^. Based on AAg ability, strains were categorized into: (1) high (HAAg, 70% AAg), (2) medium (MAAg, 20–70% AAg) and (3) low (LAAg, 20% AAg). Medium AAg ability was observed in our findings to be; 52.39% and 65.32% for *Lactobacillus acidophilus* SAM1 and *Lactiplantibacillus plantarum* SAM2, respectively. Previous research of Collado et al.^47^ reported the linkage between the aggregation ability and the cell adherence properties. Todorov et al.^48^ stated that; Co-aggregation may be important in removing pathogens from the human GIT; by preventing their attachment to the host tissue, where *Lactobacillus* strains may form a barrier to prevent pathogen colonization via co-aggregation as stated by Ferreira et al.^49^. Co-aggregation with a potential pathogen allows the probiotic strain to produce antimicrobial substances in close proximity, which may limit pathogenic strain growth in the gastrointestinal tract as mentioned by Reid et al.^50^. The varying degrees of co-aggregation of LAB isolates with different pathogens demonstrated that co-aggregation potential is a unique feature that varies depending on the type of probiotic strain and the pathogens as deduced by Cao et al.^51^. Isolates in the current research model had a remarkable potential for *S. aureus* and *C. albicans* co-aggregation. Co-aggregation and AAg have been found to be directly related properties. The results were in agreement with the previous reports of Razdan et al. (2012) and Balakrishna (2013)^13,52^. Differences in co-aggregation and AAg levels may be explained by the nature of probiotic/pathogenic cell surface components as listed by Bajaj et al. (2019) and Kumar Bajaj et al. (2014)^4,5^. Bacteria with more hydrophobic surfaces are more receptive to milk fat and aroma compounds^53^. Our strains had different high surface hydrophobicity (SHb) in different solvents. Adherence to intestinal epithelium is an important criterion for probiotic selection because it aids in colonization of the GIT, which is required for beneficial effects to be exerted^4^. Bacterial adhesion is defined by cell surface hydrophobicity (SHb), which is observed by the ability of bacteria to adhere to hydrocarbons. Overall SHb of our isolates for ethyl acetate and chloroform appears to be much higher than for xylene, which were in consistence with previous reports stated by Perez Ibarreche et al. (2014) and Bajaj et al. (2015); who showed that LAB strains revealed a greater adhesion to the basic solvent ethyl acetate than to the acidic solvent chloroform^54,55^. Our experiments deduced that; *Lactobacillus acidophilus* SAM1 was susceptible to all tested antibiotics, except; Vancomycin and Cefoxitin, while *Lactiplantibacillus plantarum* SAM2, could resist all the antibiotics used in this test. Coppola et al.^56^ was in agreement with our results concerning tetracycline sensitivity. However, this can be attributed to *L. acidophilus* is a mutant, or a higher concentration of the antibiotic may be used. Our investigations illustrated the great ability of *Lactobacillus acidophilus* SAM1 for lipase production, while, *Lactiplantibacillus plantarum* SAM2, showed a low ability for protease and amylase production. These were in accordance with what were mentioned in the studies of Zhi-gang et al. (2014) and Bogale and Prapulla (2015), in which; the ability of probiotics to produce hydrolytic enzymes such as amylase, lipase, and protease is desirable because these enzymes aid in digestive tract metabolism, this property was determined qualitatively by observing clear halo zones around colonies on MRS agar medium plates and many LAB isolates demonstrated varying degrees of amylase production capability^57,58^. Also, Tallapragada et al.^24^ stated that various strains of *Lactiplantibacillus plantarum* produced the most amylase, which was also supported by what was noticed by Garcia-Cano et al.^59^, in which, *Lactobacillus sp.* G3 4 1TO2 produced the most amylase with an average diameter of 1.23 mm of halo zone with additional observations concerning high lipolytic and proteolytic activity by *L. plantarum* OSU-PECh-A and *L. plantarum* OSU-PECh-BB. Sato et al.^60^ reinforced our observations; by detecting the lipolytic activity of *Lactobacillus casei* L-7, *Lactobacillus acidophilus* L-14, *Lactobacillus plantarum* L-34, and *Lactobacillus helveticus* L-53.

Original carbon source (Glucose) in current experiments has an enhancing effect on the growth of *Lactobacillus acidophilus* SAM1 and *Lactiplantibacillus plantarum* SAM2. This was in accordance with the investigations reported by Chen et al.^61^ who mentioned that there were significant differences between various carbon sources on the growth of *Lactobacillus acidophilus*. While, whey powder and glucose showed a noticeable good effect on the growth of *Lactobacillus acidophilus*. However, Maltose could result in a maximum production level of *Lactobacillus plantarum* ST23LD antimicrobial agents as mentioned by Todorov and Dicks (2006) and Mitra et al. (2007)^62,63^.

Our investigations in this study showed that yeast extract when used as a nitrogen source can led to the maximum rate for growth of our isolated strains. Similarly, Chen et al.^61^ reported that, organic nitrogen source could promote better growth of *Lactobacillus acidophilus* than inorganic nitrogen source. Their results showed that yeast extract and peptone were ideal nitrogen sources of *Lactobacillus acidophilus*^61^. Finally, the results of the present investigation collectively indicate that the *Lactobacillus acidophilus* SAM1 and *Lactiplantibacillus plantarum* SAM2, could be characterized as probiotic strains naturally occurring in the human mother milk, and cottage cheese as not prepared using human milk; respectively. Moreover, SAM1 and SAM2 showed potential and promising antimicrobial activities; therefore, can indicate the possibility of using the two isolates in therapeutic and prophylactic fields as natural and biological sources of antimicrobial substances, with the ability to produce different extracellular enzymes as amylase, lipase and protease. Consequently; the two isolates could share in many different industrial fields.

## Supplementary Information

Supplementary Figure 1.

Supplementary Table 1.

Supplementary Table 2.

## Data Availability

The datasets generated and/or analysed during the current study are available in the GeneBank (NCBI-Nucleotide Database) repository as following: 1.Lactobacillus acidophilus strain SAM1 16S ribosomal RNA gene, partial sequence under accession number ON495959.1 (Link: https://www.ncbi.nlm.nih.gov/nuccore/ON495959) 2.Lactiplantibacillus plantarum strain SAM2 16S ribosomal RNA gene, partial sequence under accession number ON527739.1 (Link: https://www.ncbi.nlm.nih.gov/nuccore/ON527739).
