# Supplementary material for: Identification of Lactobacillus strains from human mother milk and cottage cheese revealed potential probiotic properties with enzymatic activity
Source: Sci Rep. 2022 Dec 29;12:22522. doi: 10.1038/s41598-022-27003-2 (PMC9800376; doi:10.1038/s41598-022-27003-2)
Supplement: Supplementary file 1 — Supplementary Figure 1. [file 41598_2022_27003_MOESM1_ESM.pptx]

## Slide 1
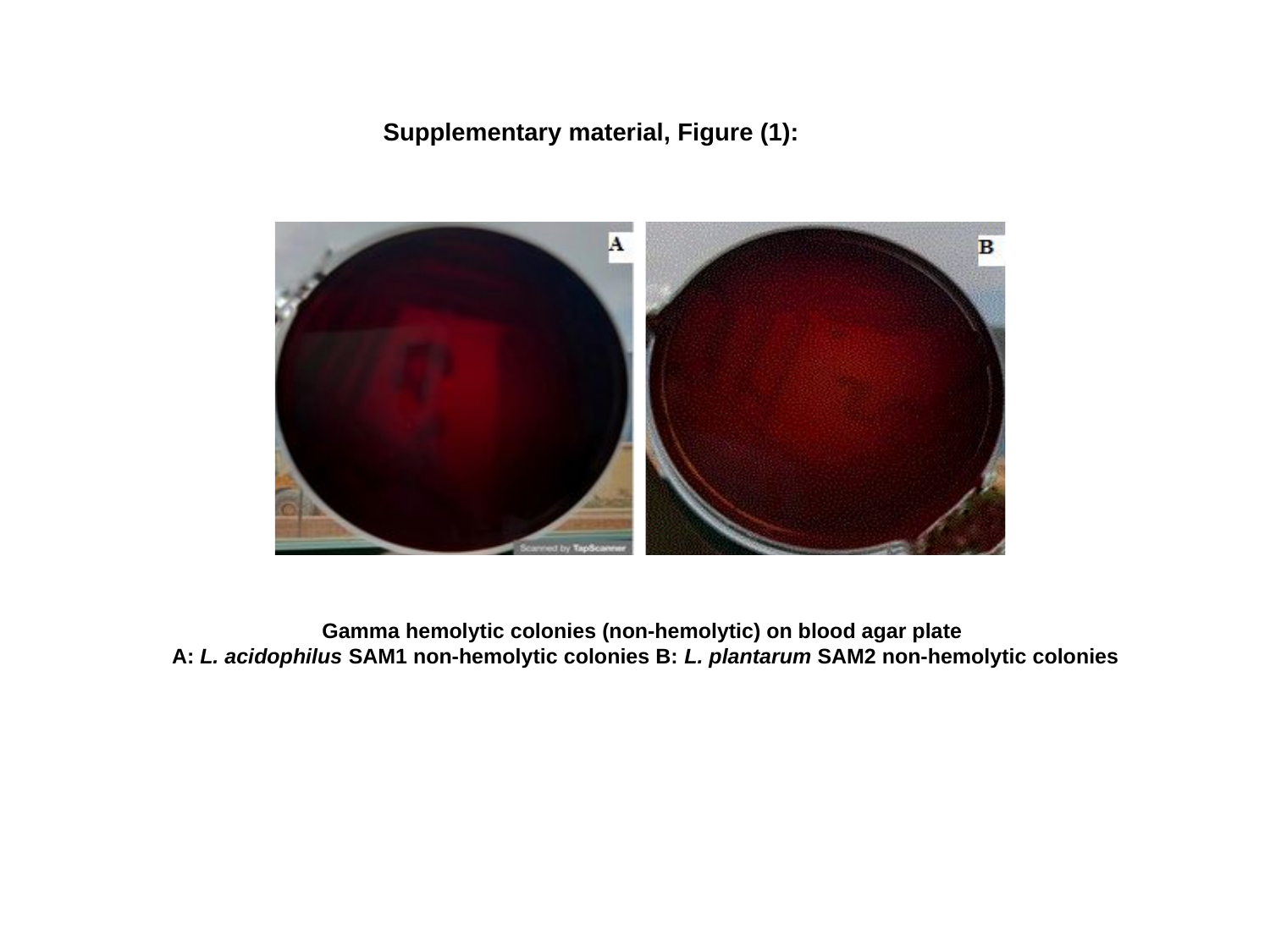

Supplementary material, Figure (1):
Gamma hemolytic colonies (non-hemolytic) on blood agar plate
A: L. acidophilus SAM1 non-hemolytic colonies B: L. plantarum SAM2 non-hemolytic colonies
