# Supplementary material for: Identification of Lactobacillus strains from human mother milk and cottage cheese revealed potential probiotic properties with enzymatic activity
Source: Sci Rep. 2022 Dec 29;12:22522. doi: 10.1038/s41598-022-27003-2 (PMC9800376; doi:10.1038/s41598-022-27003-2)
Supplement: Supplementary file 2 — Supplementary Table 1. [file 41598_2022_27003_MOESM2_ESM.docx]

**Supplementary material**

**Table (1): Morphological and biochemical characterization of the LAB strains**

| **Parameter** | ***Lactobacillus acidophilus* SAM1** | ***Lactiplantibacillus plantarum* SAM2** |
| --- | --- | --- |
| **Cell morphology** | Rods | Rods |
| **Cell arrangement** | In pairs and short chains | Long slender rods in chains |
| **Gram stain reaction** | + | + |
| **Catalase activity** | - | - |
| **Oxidase test** | - | - |
| **Indole test** | - | - |
| **Methyl red test** | + | - |
| **VP test** | - | - |
| **Citrate utilization** | - | + |
| **Arginine** | - | - |
| **Motility** | Non motile | Non motile |
| **Endospore formation** | Non-sporing | Non-sporing |
| **Growth in medium with** NaCl %  2%  4%  6% | +  +  - | +  +  + |
| **Fermentation of**  Glucose  lactose  sucrose  Gas production  Acid production | +  +  +  +  + | +  +  +  -  + |
| **TSI test** | Acid/acid | Acid/acid |
| **Growth temperature**  10º C  30^o^C  45º C | -  +  + | -  +  + |
