# Supplementary material for: Identification of Lactobacillus strains from human mother milk and cottage cheese revealed potential probiotic properties with enzymatic activity
Source: Sci Rep. 2022 Dec 29;12:22522. doi: 10.1038/s41598-022-27003-2 (PMC9800376; doi:10.1038/s41598-022-27003-2)
Supplement: Supplementary file 3 — Supplementary Table 2. [file 41598_2022_27003_MOESM3_ESM.docx]

**Supplementary material**

**Table (2): Susceptibility spectrum of *L. acidophilus*** **SAM1, and *L. plantarum*** **SAM2** **toward different antibiotics.**

| **Antibiotic** | **Concentration** **(μg/disc)** | **Degree of sensitivity***  *L. acidophilus* SAM1 | **Degree of sensitivity***  *L. plantarum* SAM2 |
| --- | --- | --- | --- |
| **Ampicillin** | 10 | S  (1.2 cm) | R |
| **Penicillin** | 10 | S  (2.8 cm) | R |
| **Erythromycin** | 15 | S  (1.9 cm) | R |
| **Ciprofloxacin** | 10 | S  (1.5 cm) | R |
| **Tetracycline** | 30 | S  (1.7 cm) | R |
| **Vancomycin** | 30 | R | R |
| **Cefoxitin** | 30 | R | R |

*: (S) Sensitive, (R) Resistant
